# How biological background assumptions influence scientific risk evaluation of stacked genetically modified plants: an analysis of research hypotheses and argumentations

**DOI:** 10.1186/s40504-017-0057-7

**Published:** 2017-08-14

**Authors:** Elena Rocca, Fredrik Andersen

**Affiliations:** School of Economics and Business, Norwegian University of Life Sciences, P.O. Box 5003N, -1432 Ås, Norway

## Abstract

Scientific risk evaluations are constructed by specific evidence, value judgements and biological background assumptions. The latter are the framework-setting suppositions we apply in order to understand some new phenomenon. That background assumptions co-determine choice of methodology, data interpretation, and choice of relevant evidence is an uncontroversial claim in modern basic science. Furthermore, it is commonly accepted that, unless explicated, disagreements in background assumptions can lead to misunderstanding as well as miscommunication. Here, we extend the discussion on background assumptions from basic science to the debate over genetically modified (GM) plants risk assessment. In this realm, while the different political, social and economic values are often mentioned, the identity and role of background assumptions at play are rarely examined. We use an example from the debate over risk assessment of stacked genetically modified plants (GM stacks), obtained by applying conventional breeding techniques to GM plants. There are two main regulatory practices of GM stacks: (i) regulate as conventional hybrids and (ii) regulate as new GM plants. We analyzed eight papers representative of these positions and found that, in all cases, additional premises are needed to reach the stated conclusions. We suggest that these premises play the role of biological background assumptions and argue that the most effective way toward a unified framework for risk analysis and regulation of GM stacks is by explicating and examining the biological background assumptions of each position. Once explicated, it is possible to either evaluate which background assumptions best reflect contemporary biological knowledge, or to apply Douglas' 'inductive risk' argument.

## Introduction

### Background

The increased use of technology for resource production creates new uncertainties concerning human health and environmental safety. Decision-making in the governance of new technologies relies heavily on scientific risk assessment. However, this reliance has been debated due to its numerous limitations (Jasanoff 2005), one of the problems being that risk assessment is under-determined by evidence (Miller and Wickson 2015). Accordingly, instances of diverging evaluations of risk are frequently justified by the same evidence. A striking example is the recent assessment of carcinogenicity of the herbicide glyphosate by two different agencies. IARC (International Agency for Research on Cancer) classified glyphosate as a “probable human carcinogen”, while shortly after EFSA (European Food and Safety Agency) concluded that “glyphosate is unlikely to pose a carcinogenic hazard to humans” (Portier et al. 2016). This and similar cases illustrate how risk evaluation includes extra-evidential components (Sawyer 2015). One main topic of this discourse has been that, although social dynamics, value judgements, premises and assumptions play an important role in shaping risk evaluation, they usually remain hidden from regulatory scrutiny (Hartley et al. 2016; Wickson and Wynne 2012). In the last decades, it has been argued that debates stemming from diverging risk evaluations can be improved when evaluators state their non-epistemic values (ethical, social, and political) explicitly. Objectivity then follows from transparency about, rather than absence of, value judgements (Longino 1990; Douglas 2000; Althaus 2005; Hermansson 2012; Hartley et al. 2016).

Here, in line with existing literature, we highlight that there are different types of extra-evidential components influencing the overall risk evaluation. The first type are the socio-political value judgements involved in the process (Douglas 2000; Longino 1990; Hermasson 2012). These are especially relevant for risk assessment, because of its embedding into the political and social realm of evidence-based policymaking. Another type of extra-evidential component are what we may call background assumptions. These are the framework-setting suppositions we all apply in order to understand some new phenomenon. Naturally, a scientist will adopt and apply different background assumptions depending on which field she is in. Additionally, which will be the main topic of our discussion, there are multiple scientifically respectable sets of background assumptions within a single field, and especially so if the field in question is under development. Although all scientific research involves the application of background assumptions, these are mainly discussed in relation to basic science (see for instance Stump 2015, Galilei 1615, Reichenbach 1927, Kuhn 1973, Einstein 1936).

There are ongoing debates in the philosophy of science concerning the nature of background assumptions in basic research and their origins. Are they “free creations” as suggested by Einstein (1936), conventions as suggested by Reichenbach (1927), or empirically derived as argued by for instance Pap and Stump (see Stump 2015)? There is, however, agreement across the board concerning the functional aspect of background assumptions. Background assumptions are more general than new evidence, and play a regulatory function in relation to it. This means that, whatever their origin, background assumptions co-determine how a scientist chooses methodology, analyses data, and picks out relevant evidence. In short, background assumptions are the lens through which we view new information. We shall focus on this regulatory function and leave the question of origins open. Scientists operating within the same field but with different background assumptions might disagree on the overall rules of scientific inquiry as well as its content.

It is uncontroversial that different fields of basic research are often lacking, and would benefit from, an explication of background assumptions behind common concepts. In biology, for instance, Carver et al. (2008) find that articles and textbooks related to gene technology frame the notion of gene by using different metaphors than articles and textbooks related to evolutionary and environmental biology. For gene technology, the gene concept is typically illustrated as prone to isolation and manipulation. For evolutionary and environmental biology, it is dynamic and interactive. Stotz et al. (2004) find that different conceptualizations of gene in different areas of biology follow from the use of different experimental practices. Importantly, the single scientist might not always be aware that her background assumptions are debatable since such assumptions typically go unopposed and only implicitly enter the scientific discourse. Kuhn even argued that ignoring and omitting alternative approaches is intrinsic to the scientific process (Kuhn 1970).

In basic science, key figures such as Einstein and Galilei not only recognized that background assumptions make up the basic “rules of the game” (Einstein 1936). They also crucially indicate that, once explicated, disagreements are resolvable (Galilei 1615; Galilei 1632) It is our contention that this applies also in the case of scientific risk-evaluation.

Scientific risk-evaluation of biotechnology is an applied field of research and thus the relevant background assumptions can be expected to be of a narrower variety than in basic research. We exclusively treat those assumptions pertaining to biology and refer to them as *biological background assumptions* throughout.

### Aim of the article

In this article, we are extending the discussion over background assumptions from the basic sciences, where it is well established, to the scientific and regulatory debate over genetically modified (GM) plants. While the role of political, social and economic values is often mentioned in the debate over GM plants risk assessment (see for instance Wickson and Wynne 2012), to our knowledge the identity and role of biological background assumptions are only implicitly acknowledged. We suggest that this is detrimental for at least two reasons. First, it hinders effective communication between opposing camps. An example is found in a recent comment from the Norwegian Science committee for Food Safety (VKM) to an application for the introduction of a variety of genetically modified maize (VKM, 2016). VKM remarks that certain claims in relation to risk in the application are not substantiated, the underlying problem being that there are different ways to understand what constitutes proof. In this particular case, the applicant provides proof from argument, while the VKM requires proof from experiment. Problems such as “what constitutes proof of food and feed safety” rely heavily on a scientist’s biological background assumptions. In relation to food and feed risk assessment, these can concern experimental set-up, generalizability of behaviors of biological entities across contexts, relations between scientific methods and biological processes and so on. If neglected, experts holding different biological background assumptions easily misunderstand each other as they attach different meanings to central concepts.

A second, crucial point is in connection to Douglas’ influential argument of ‘inductive risk’ (Douglas 2000). Since diverging biological background assumptions are often all scientifically justifiable, and since the consequence of adopting one over the other might have an impact on the overall risk evaluation and therefore on decision making, the choice must imply extra-scientific considerations. By requiring an ethical - social commitment, this argument clearly implies a conscious, active choice by the scientist: “In making a choice between these positions, scientists must consider the consequences of their choice, particularly if they are wrong” (Douglas 2000, p. 576).

Such a choice, we argue here, is not yet available in many debates over GM plants safety. As we show in our case study, the biological background assumptions in certain debates remain un-explicated and thus proponents of either position argue as if there were no alternatives to their theoretical approach. For instance, it is commonly argued that dissenting argumentations have “no scientific basis” (Kok et al. 2014, p. 72). If our analysis of the debate in the case study is correct, we should not expect a definite resolution merely from production of new evidence. Rather, there is need for a debate over issues that are more basic. Given contemporary knowledge of biology, are the alternative sets of biological background assumptions equally scientifically justifiable? If they are, which degree of uncertainty is involved in the choice of one over the other? If there is no clear scientific preference of one set of biological background assumptions over the other, the discussion can be enlarged from strictly technical to philosophical, for instance by asking if there are epistemic advantages to one set of biological background assumptions over another (coherence, simplicity, explanatory power, etc.). Finally, by moving to non-epistemic consideration, arguments like Douglas’ inductive risk apply.

Our contention is that the time is ripe for an explication and analysis of the active biological background assumptions in the field of risk assessment of GM plants. Indeed, a full explanation of experts disagreement needs an elucidation of *all* its extra-evidential components, both value judgements *and* biological background assumption, since these two are intimately connected (Longino 1990, Douglas 2000). As a case study, we focus on those biological background assumptions that are relevant for the safety assessment of stacked GM plants (GM stacks).

### GM (single), GM stacks, and conventional hybrids. The debate over risk assessment

For millennia, farmers and breeders have crossed naturally occurring plants in order to produce hybrid plants with desired traits. This practice is generally considered to produce few to no issues concerning food and feed safety. Thus, the globally agreed upon safety assessment regime concerning conventional hybrids is non-rigorous.

More recently, biotechnological development facilitated the construction of GM plants. These are produced by introducing a gene fragment from one species (often a bacterium or another plant species) into the genome of a conventional plant. The gene fragment that is introduced is referred to as a transgene, and the protein it produces is a transgenic protein. The main aim of this technology is to introduce a new and advantageous trait to the conventional plant without interfering with its domestic traits. For instance, Roundup ready soybean is obtained by inserting a version of the EPSP gene, originally produced by *Agrobacterium strain CP4*, into a soybean plant. The CP4 EPSP transgenic protein, expressed by the GM soybean plant, confers resistance to the herbicide Roundup™ (Funke et al. 2006). There is global agreement that a single GM plants safety must be assessed before it is introduced in the market. Such safety assessment includes molecular and phenotypical characterization, food/feed risk assessment and environmental risk assessment (Codex Alimentarius Commission (CAC) 2003).

A more recent development of GM plant technology are *stacked* GM plants (GM stacks), which carry two or more transgenic modifications (Que et al. 2010). The GM stacks object of this paper are obtained by first producing two or more GM plants, each containing a single transgene, and then crossing them by using the techniques of conventional breeding.^1^ A GM stack thus contains two or more transgenes in distinction to a GM (single) plant, which contains only one. There is no global agreement concerning how to assess the safety of GM stacks (Pilacinski et al. 2011). This lack of agreement can be traced back to an underlying lack of agreement concerning what a GM stack is.

On the one hand, it is possible to think of a GM stack as a novel GM entity. This is the general thinking for instance in European safety assessment agencies. As reported by the European Commission Directorate General for Health Consumers, European legislation embraces the rationale that “a stack of two GMOs is simply another distinct GMO, a ‘new’ entity” (European Commission Directorate General for Health and Consumers, 2010). Thus, European agencies require data on stability of the inserts, level of expression of the transgenic events, potential interactions between events, comparative analyses of nutritional composition and agronomic traits (European Food and Safety Agency (EFSA), 2007).

On the other hand, one could argue that since the GM stacks are obtained through traditional breeding techniques, they are no more biologically novel than any other hybrid obtained in the same manner. Thus, GM stacks should be regulated as the conventional hybrids. Accordingly, regulatory agencies countries such as USA, Canada and Australia apply a less rigorous risk assessment scheme before introducing GM stacks on the market (Pilacinski et al. 2011).

A central aspect of the disagreement between the European and American regimes is the question of whether one can infer knowledge concerning the safety of a GM stack from knowledge concerning the safety of its parental GM (single) plants. In the American regime, for instance, such an inference is granted and additional testing is required only in cases where one can reasonably expect that the transgenes and their products will interact (Pilacinski et al. 2011).

Within the scientific community, argumentation has been offered both in favor and against the simplification of GM stacks regulation following the American model (Agapito-Tenfen et al. 2014; Ali et al. 2014; Kok et al. 2014; Kramer et al. 2016; Londo et al. 2011; Mesnage et al. 2013; Steiner et al. 2013; Weber et al. 2012). Based on the same scientific evidence, scientists disagree on whether GM stacks are new GM plants, requiring new evidence for their risk assessment, or whether information about their risk can be inferred from the evidence provided during the risk assessment of the parental, GM (single) plants.

## Research hypothesis

Our research hypothesis is that there are diverging sets of biological background assumptions in the ongoing scientific debate concerning whether GM stacks are novel transgenic entities or not. If this is true, it helps explain the plurality of risk assessment regimes that exist at the moment. Moreover, it is our contention that if our hypothesis is corroborated, and a part of the disagreement lies at the level of biological background assumptions, a fruitful debate over the risk assessment of GM stacks requires a debate at this level.

In order to test our hypothesis, we reviewed and analyzed the peer-reviewed literature dealing with risk evaluation of GM stacks. The aims of the analysis were: (A) to identify implicit biological background assumptions that underlie the scientific argumentation as well as research hypotheses about risk of GM stacks, and (B) to test whether differing biological background assumptions are central to the opposing positions.

## Method

Literature was searched in PubMed using the keywords “stacked GMO”, “stacked transgene”, “risk assessment” and “uncertainty”, and selected using the following criteria. (a) Type of argument: only scientific papers were included. Socio-economic and practical issues such as feasibility, length and costs of risk assessment fall outside the purposes of our analysis. (b) Publication date: our aim was to compare papers based on common evidence, therefore older publications (before 2011) were excluded. (c) Transparency: selected papers stated explicitly their standpoint in respect to the issue of GM stacks risk assessment (Table 1). (d) Specific topic: in order to narrow the selection, we included only papers dealing with molecular composition and stability of GM stacks in relation to food/feed safety.

**Table 1:** Classification of analyzed literature

Paper	Classification	Quote
Weber et al., 2012	Standpoint 1 (S1)	“Evaluating transgenic insertion stability in a GE stack does not provide information that can contribute to its safety assessment”
Steiner et al., 2013	Standpoint 1 (S1)	“If the events are unlikely to interact, no additional assessment should be needed to make a safety determination for the GE stack, because each individual event has already undergone extensive independent safety assessments”
Kok et al., 2014	Standpoint 1 (S1)	“… There is no sound scientific argument to require full dossiers for stacked GM event varieties that comprise single events that have already been elaborately assessed”
Kramer et al., 2016	Standpoint 1 (S1)	“An alternative food and feed risk assessment strategy for stacked GM events is suggested based on a problem formulation approach that utilizes (i) the outcome of the single event risk assessments, and (ii) the potential for interactions in the stack, based on an understanding of the mode of action of the transgenes and their products”
Londo et al., 2011	Standpoint 2 (S2)	“Understanding the potential fitness costs and benefits of combining transgenic traits in plant species is necessary to properly address impacts of crop production”
Mesnage et al., 2012	Standpoint 2 (S2)	“Potential side effects of combined pesticides residues should be assessed”
Ben Ali et al., 2014	Standpoint 2 (S2)	“Since stacked events contain multiple viral promoters the susceptibility to instabilities may be increased”
Agapito-Tenfen et al., 2014	Standpoint 2 (S2)	“GM plants containing stacked events cannot be considered generally recognized as safe without specific supporting evidence”

Selected papers (eight in total) were classified, according to explicit statements, as belonging to two opposite standpoints. Papers classified as *standpoint 1* (S1) argued that risk evaluation about stability and potential interactions in GM stacks can be inferred from the risk assessment of the parental GM (single). Papers classified as *standpoint 2* (S2), on the contrary, claimed that some issues cannot be inferred from the risk assessment of parental GM (single) and require the generation of new evidence. Following these criteria, four papers were classified as S1 and four as S2 (Table 1).

## Results

### Identification of biological background assumptions

As a result of our analysis, we found that arguments in the selected papers rely on two types of premises: openly stated premises, and implicit, unstated premises. In every argument, both kinds were necessary steps toward the stated conclusions.

We diagnosed essential, unstated premises as biological background assumptions. Our main finding is that these are necessary in order to give a full account of each standpoint. Moreover, we found that neither standpoint can be rigorously defended without reference to the biological background assumptions.

S1 papers argue that (i) in relation to risk, GM stacking is equivalent to conventional breeding of conventional plants. A biological background assumption in this argument is that equivalent biological processes follow when equivalent techniques are applied. We have called this *equivalence of biological process*. Furthermore, S1 papers argue that (ii) one can derive knowledge of GM stacks from knowledge of the GM (single) parental plants. This argument relies on the biological background assumption that genes and their products behave equivalently in parental GM (single) and in GM stacks, an assumption we have called *equivalence of entity behavior*.

S2 papers argue that (i) in relation to risk, there are potential differences between GM stacking and conventional breeding of conventional plants. This argument relies on the further supposition that biological processes of GM stacking unfold differently from those of conventional breeding, since the techniques applied are identical. We have called this *variability of biological process*. S2 papers also argue that (ii) genes and their products might behave dissimilarly in parental GM (single) and GM stacks, thus implying that the same biological entities behave differently across contexts. We have called this *variability of entity behavior.* 2

In both S1 and S2 papers we find that the general arguments presented are dependent on these ontological commitments. For an overview of the analysis of S1 papers, including argument overviews, biological background assumptions, and relevant evidence, see Table 2. For an analysis of S2 papers, including research premises, relevant evidence, biological background assumptions, research hypotheses and aim of the study, see Table 3 (all S1 papers we identified were argumentative, while all the S2 papers are research papers; the two tables therefore have slightly different organization).

**Table 3:** Analysis of papers supporting standpoint 2 (S2)

Author	Premise(s)	Relevant evidence, or lack of relevant evidence	Biological background assumptions and general hypotheses	Aim of the study
Londo et al. (2011)	The advantage of a genetic modification depends on the context where plant lives (duration and strength of selective pressure).	When a plant naturally evolves resistance to herbicide, such resistance provokes fitness cost when herbicide selection is not applied (metabolic drain). Even if one transgene is disadvantageous, its frequency can be increased by the selection of the second transgene (Hitch-hiking effect).	***P1: variability of biological process*** **H1:** Stacking transgenes could be a metabolic drain and decrease fitness: ***P2: variability of entity behavior*** **H2:** The costs of each transgene might not be additive, therefore might not be foreseeable from analysis of GM single.	Evaluating the fitness of GM stack and GM (single) lines of Canola relative to control, non-transgenic lines in a common garden environment, under different selective pressures.
Mesnage et al. (2013)	Pesticide residues and herbicides co-occur in the plants, synthetized by the plant itself (GM stack) and/or through external pesticide treatment.	Toxicity studies that check the real effects of combination of toxins are missing.	***P1: variability of entity behavior*** **H1**: Pesticides and herbicides in combination could have a real effect on human cells that is not predictable	Evaluating in a sensitive model of human cells in vitro toxicity of a mixture of Bt toxins and Glyphosate formulations
Ben Ali et al. (2014)	Stability of the genetic insert is an important aspect to ensure food feed safety. Guidelines say that the insert must not change during the cultivation and propagation.	Studies showed instances of rearrangement and alteration of the genome in GM plants during the post-release monitoring. New methods for profiling of epigenome, transcriptome, proteome have identified changes in some GM varieties compared to unmodified comparators.	***P1: variability of biological process*** **H1**: GM stacks might be more sensitive to instability and alterations of the transgene. Such alterations might not be identifiable with the low-sensitivity methods used in normal risk assessment (Southern Blot).	Identifying DNA alterations in the transgenic DNA fragment of GM plants, particularly in GM stacks. Evaluating the impact of the identified mutations in the aminoacid sequence of the transgenic proteins
Agapito - Tenfen et al. (2014)	Compositional and nutritional comparison between GM stacks and GM (single) might not be fit to reveal unintended effects	There is no comparison between molecular characterization of GM stacks and GM (single)	***P1: variability of biological process*** **H1:** some effects specific to the process of stacking might remain undetected by traditional analysis. ***P1: variability of entity behavior*** **H1:** The combined transgene could provoke variations outside the range found in GM (single)	Evaluating changes of protein profiles in stacked events versus single events and control plants. Comparing the level of transgene expression in stacked events compared to single events and control plants

Legend: **P** = implicit premise (biological background assumptions). **H** = hypothesis.

As an illustration of our findings and methodology, we will describe in detail the comparative analysis of one paper for each standpoint (Steiner et al. 2013 as representative for S1 papers and Agapito-Tenfen et al. 2014 as representative for S2 papers).

### An illustration of findings and methodology

Steiner et al. (2013), promoting S1, and Agapito-Tenfen et al. (2014), promoting S2, both present a two-part argument. One part considers potential risk in relation to methodology. Here, the central issue is whether GM stacking induces any change in relation to genetic stability. The main element of disagreement is whether stacking two or more transgenes by crossing single GM plants and selecting the transgenic traits, significantly affects the overall stability of the resulting plant.

The second part of the argument considers risk and the possibility of prediction. A core issue being whether one can make inferences concerning how transgenic products will interact with each other as well as with the domestic proteins in a GM stack, from available information of such interaction in a GM (single) parental plant.

#### Risk and methodology

The two papers start from a common ground of two assumptions:

- Conventional breeding brings about a large number of new and unknown variations at molecular level, yet it hardly ever creates any novel risk or hazard.
- It is known (from the risk assessment of parental GM (single) plants) that the detected level of variation in the phenotype^3^ when crossing conventional plants with each other is the same as when crossing a GM (single) plant with a conventional plant.

From these common premises, the two papers argue along opposite routes. Steiner et al. (2013) argue that GM stacking is equivalent to conventional breeding, and suggests that as far as risk is concerned, there is no difference between GM stacks and conventional hybrids. Furthermore, it is argued that GM stacking produces no novel safety issues in relation to the GM (single) parental plants. Hence, GM stacking adds no risk relative to conventional breeding.

Agapito-Tenfen et al. (2014) argue the opposite position. As there could be “unique effects of stacking two or more genetic inserts”, these could bring about unpredictable variation in the whole plant physiology. GM stacking could therefore differ from conventional breeding. However, it is argued, this unpredictable variation could go undetected by the standard breeding methodology of phenotypic selection and comparison. Furthermore, comparisons of GM (single) plants and GM stacks are scarce. Hence, knowledge about the similarity between GM single plants, conventional hybrids, and GM stacks is too limited to make any general conclusion concerning risk and safety.

Starting from the same evidence, how can we end up in such diverging positions? Both arguments start from a common set of premises stating that: P1) breeding always produces variations; P2) conventional breeding processes produce no novel food or feed safety issues; P3) crossings between GM (single) plants and conventional plants produce no detected novel food or feed safety issues, and P4) in (i) conventional breeding, (ii) crossing GM (single) with conventional plants, and (iii) GM stacking, the exact same methodology is applied (phenotype selection).

There are two central elements running through these premises. One, found in premise 4, concerns the similarity in methodology. The other, found in premise 1, 2, and 3, concerns the underlying biological processes, i.e., the plant being safe or unsafe for consumption. On their own, these premises are not sufficient for concluding one way or the other. One could argue that the methodology guarantees the safety of conventional hybrids, and thus that the similarity in methodology guarantees food and feed safety in all instances. However, one could also argue that these are separate issues and that the safety of conventional hybrids follow from some evolutionarily developed mechanism. If so, we do not know how this mechanism will cope with multiple transgenic inserts. In other words, further premises are needed in order to conclude either way.

Steiner et al. (2013) argue directly from the safety of conventional hybrids to the safety of GM stacks. “Crossing different non-[GM] genotypes results in novel gene combinations and, in turn, novel interactions, but no safety issues have been identified. Hence, this process^4^ of creating novel interactions via crossing is highly unlikely to be influenced by the stacking of transgenes” (p.1589). This argument rests on the further premise that as long as the methodological procedure is identical, the underlying biological process will unfold in a similar manner. This is the biological background assumption of the *equivalence of biological process* that we introduced earlier. Only under this assumption do the above premises lead to the conclusion of Steiner et al. This, in and of itself, does not speak against the argument. Rather, it indicates that a possible resolution of the debate might be found by questioning the biological background assumption and possibly finding a way to test it empirically.

As long as the *equivalence of biological process* is accepted, stacking a couple of transgenic inserts into a whole genome, already containing thousands of recombination hotspots, appears innocuous. If the only significant difference between conventional breeding, crossing between GM (single) and conventional plant, and stacking GM (single) plants is the presence of none, one or two transgenic inserts, it can be argued that such variation is not likely to introduce big changes in the process. Therefore, why should we not infer safety from conventional breeding to stacking? Steiner et al. (2013) concludes that, methodologically, stacking single GM plants produces no novel food and feed safety issues. However, if the *equivalence of biological process* assumption is denied, or simply doubted, one can argue for the opposing position. Agapito-Tenfen et al. (2014) follow this latter route and propose that “GM plants containing stacked events cannot be considered generally recognized as safe without specific supporting evidence” (p. 348).

Demanding specific evidence does not make sense under the biological background assumption of equivalence of biological process. The benefit of a biological equivalence of process is exactly that we can make general claims without specific data. Denying this possibility, is denying the biological background assumption itself. Agapito-Tenfen et al. (2014) therefore need a further premise in order to reach their conclusion. This premise is the alternative assumption of *variability of biological process*, which implies separating the methodological similarity from any claims of biological equivalence.

For scientists promoting S2 therefore, even if selection of the most stable phenotype guards the stability (and thus safety) of conventional crop and single GM plants, the same is not warranted in the case of stacked GM plants. Some relevant process-specific effects might be orthogonal to phenotypical stability as standardly evaluated, and might therefore remain undetected. Since the whole process (and not only the presence of transgenic inserts) is seen as potentially distinct, such possible undetected effects “might have an effect on the plant metabolism and physiology” (ibid, p 348), encompassing the mere presence of transgenic inserts but not ending with it. Agapito-Tenfen et al. (2014) therefore conclude that GM stacking might provoke novel food and feed safety issues.

#### Risk and prediction

The diverging arguments that follow from *variability or equivalence of biological process* lead to further divergence concerning whether one can draw conclusions about the resultant GM stack from previous tests of the GM (single) parental plants. From the perspective of *equivalence of biological process,* Steiner et al. (2013) can argumentatively treat the transgenes and their products in relative isolation. For, if biological processes evolve similarly across contexts, the entities involved in these processes will also behave similarly. This implication is central to Steiner et al. (2013) as they argue that one can infer the behavior of transgenic proteins in a GM stack, from knowledge of their behavior in the parental GM (single) plants.

Only in the specific case in which “(…) a plausible hypothesis can be developed for an interaction that may affect either food or feed safety, then further questions should focus on the likelihood, nature, and significance of the interaction.” (ibid p. 1590). Predictions about the behavior of transgenic proteins in stack GM can be made by asking questions such as: is there a physical interaction between the two proteins, so that they could form hetero-polymers with unique activity? Do the proteins interact with common metabolic pathways? Are they expressed simultaneously and in the same cell compartment? Could the combination create new metabolic pathways in the stacked GM? And so on.

In order for this line of reasoning to be conclusive, one must first suppose that transgenes and their products behave comparatively in a GM (single) plant and in a GM stack. Inferences from one to the other is only valid if one grants this supposition, which is the biological background assumption *equivalence of entity behavior*. If we suppose equivalence of entity behavior regardless of the number of transgenic insertions in the plant background, we can also suppose knowledge of the effects of transgenic inserts in a GM stack from tests performed on the GM (single) parental plants. In total therefore, we can suppose knowledge of how the process will unfold as well as how the transgenes and their products will behave, if we suppose the biological background assumption. Then, our only remaining lack of knowledge concerns what will happen if the transgenic inserts somehow influence each other. Steiner et al. (2013) conclude that the only remaining question is whether products of the events will interact with each other. Again, this argument only holds under the biological background assumption that there is *equivalence of biological process* as well as *equivalence of entity behavior*. The contrary assumption, *variability of biological process,* suggests that one cannot isolate interactions between two transgenic products from interactions of each transgenic product with domestic proteins. Rather, on this biological background assumption, *any* interaction between transgenic products and domestic proteins might influence how the transgenic products behave and interact among each other.

*Variability of biological process* denies the possibility of keeping related variables pertaining to such interactions under isolated control. Therefore, variability of biological process also dictates that when the overall biological process varies, no conclusion can be drawn concerning the behavior of a single entity. Agapito-Tenfen et al. suppose that (i) conventional breeding, (ii) crossing GM (single) with conventional plants, and (iii) GM stacking involve different biological processes and that the behavior and effect of an extra transgenic insert remains largely unknown. In effect, Agapito-Tenfen et al. (2014) suppose the *variability of entity behavior* and therefore argue that, even if we know the behavior of transgenes and their products in the GM (single) parental plants are known, we do not know their behavior in a GM stack. “Literature on molecular characterization of GM stacked events is scarce, and the comparison of their expression levels and potential cellular interaction to parental single GM lines is absent… Hence, there is lack of data of the kind that might be important in order to reliably assess the safety of stacked GM.” (p. 360). And further: “…compared to parental single event varieties … genome changes in stacked GM Maize may influence the overall gene expression … [There are] possible synergistic and antagonistic interactions following transgene stacking into the GM maize genome by conventional breeding” (p. 360). In total therefore, we do not know how the biological process of GM stacking unfolds, and we cannot conclude from potentially different processes how the relevant entities will behave.

So far, we identified and illustrated two sets of biological background assumptions underlying two opposing lines of argument. For clarity, we have determined separate ontological commitments concerning issues that are intimately connected and closely dependent on each other (processes and entities). Although we treat them here as separate ontological commitments, they may well be instances of the same background assumption. We also showed how such assumptions are necessary premises of the stated arguments. In the next paragraph, we describe how biological background assumptions affect evaluations of methodology.

### Biological background assumptions and methodology

The demand for relevant empirical evidence obtained through an appropriate methodology is one of the demarcations of science as opposed to mere speculation (Ellis and Silk 2014). In basic research, it is acknowledged that a scientist’s evaluation of which methodology is appropriate for a particular set of phenomena depends, at least partially, on her background assumptions concerning the realm of inquiry. If a theoretical framework changes, including background assumptions, the impact and relevance of a certain methodology and relative evidence change with it (see Einstein 1905, Galilei 1632 and for an example in biology Balkwill and Mantovani 2001) .^5^ In risk evaluation of GM stacks, one should expect that scientists with opposing biological background assumptions would disagree on the choice of appropriate methods of enquiry. We have already argued that proponents disagree on whether risk related inferences from parental GM (single) to GM stacks are appropriate. In the following, we shall see that proponents of S1 and S2 disagree on methodology in general. Most prominently, they disagree on the appropriateness of Southern Blotting as an exhaustive method to check the insert’s stability and targeted comparative analyses as a method to detect potential hazards. For brevity, we describe only at the debate over targeted comparative analyses (See also Tables 2 and 3).

**Table 2:** Analysis of arguments in papers supporting standpoint 1 (S1)

Authors	Overall arguments	Premises *including Biological Background Assumptions*	Conclusions	Main Relevant Evidence
Weber et al., 2012 Steiner et al., 2013 Kok et al., 2014 Kramer et al., 2016	Although involving randomness, conventional breeding is a safe procedure that introduces no novel safety issues. Selection for stability is intrinsic of conventional breeding and further guarantees its safety. GM stacking process does not produce a new level of risk in respect to the risk level of parental stable GM (single).	P1) Conventional breeding of conventional plants produces no novel food and feed safety issues **P2)** E***quivalence of biological process***	GM stacking produces no novel food and feed safety issues	Safe history of use of crops obtained through conventional breeding
Steiner et al., 2013 Kok et al., 2014 Kramer et al., 2016	Risk assessment of parental GM (single) includes holistic analyses, such as phenotypic, nutritional, compositional comparison between the GM (single) plants and their unmodified counterparts. Such analyses account also for the interactions between the transgenic components and the rest of the plant (domestic components). These interactions result in a GM (single) that can be bred with a conventional variety without provoking change in the phenotype, despite inherent variation (stable GM single). No new level of risk is plausible In GM (single) x GM (single) in comparison to GM (single) x conventional plant.	P1) In the parental GM (single) there are interactions between transgenic genes (and their products) and domestic genes (and their products) P2) Such interactions do not provoke any new range of variability or instability respect to conventional counterparts **P3)** ***Equivalence of entity behavior***	Transgenes and their products do not provoke any new range of variability or instability, compared to conventional counterparts, when they interact with domestic genes and their products in a GM stack.	Parental GM (single) are phenotypically stable, meaning that the cross GM (single) X conventional generates new GM (single) with phenotype comparable to the parental plant 22 GM Stacks were assessed by EFSA so far. No variation was found that is outside the range of variation in parental GM (single). Data from individual studies about single events and stacks containing two, three and four Syngenta event combinations were compared. The variation of compositional analysis and protein expression in the stacked event was mostly within the prediction intervals derived from data relative to single events.
Steiner et al., 2013 Kok et al., 2014	Since parental GM (single) are phenotypically stable, safety assessments for the parental stable GM (single) are directly applicable to the GM stack. The only remaining safety question that the individual event assessments do not address is that of interactions between the products of the combined transgenes.	P1) GM (single) plants are safe and stable. **P2)** ***Equivalence of entity behavior***	Genes and their products that were proven safe and stable in GM (single) will be maintained in the same range in GM stacks.	Risk assessment of parental GM (single)
Steiner et al., 2013 Kok et al., 2014 Kramer et al., 2016	Knowledge from risk assessment of parental GM (single) and knowledge of transgenic proteins gives valid prediction of interactions between transgenic proteins coming from different parental GM (single), and the metabolic pathways of the GM stack.	P1) Behavior of transgenic proteins in the parental GM (single) is known. P2) Metabolic pathways of the parental GM (single) are known ***P3) Equivalence of entity behavior***	Interactions between the transgenic proteins and the metabolic pathways of the GM stack are predictable.	Risk assessment of parental GM (single)
Steiner et al., 2013 Kok et al., 2014	Potential interactions between the transgenic proteins that get stacked in the same plant are predictable. The overall development of a hypothesis for such interactions relies on knowledge of the plant variety and previous characterizations of the parental GM (single).	P1) Transgenic product behaviors in parental GM (single) are known. ***P2) Equivalence of entity behavior*** P3) Transgenic product interaction in GM stack is predictable	Potential interactions between the events within the GM stack can be predicted	Risk assessment of parental GM (single)
Weber et al., 2012	Parental GM (single) derive from an artificial process of genetic transformation. Such process could provoke undesired mutations in either of the two parental GM (single) or both. However, unintended effects of such mutations have already been evaluated and excluded during the safety assessment of the GM (single). In the GM stack there will be no unintended effects due to mutations in one of both parental GM (single).	P1) There might be mutations in the genome of parental GM (single). P2) There is no unintended effect of such mutations in the parental GM (single). ***P3) Equivalence of entity behavior***	There is no unintended effect of potential mutations in the GM stack	Risk assessment of parental GM (single)

Legend: P = premise. ***P*** = implicit premise (biological background assumption)

S1 papers take their que from previous assessments of conventional hybrids and GM (single) in combination with targeted comparative analyses of GM stacks and GM (single) plants. Comparative analyses have indicated that the gross structure of the transgenic insert as well as the variations in agronomic traits, nutritional composition and transgenic protein expression were in the same range for all the included varieties and environments (Kok et al. 2014; Kramer et al. 2016). This evidence is, in the words of Kramer et al. (2016), compelling for the conclusion that it is time to stop the rigorous testing of GM stacks and start treating them as conventional hybrids.

S2 proponents argue that the available comparisons between GM stack and GM (single) are insufficient for drawing any general conclusion about the risks involved in GM stacking. Thus, although Kok et al.(2014) and Kramer et al. (2016) report instances of GM stack analyses where levels of variation remain well within limits of safety, no general conclusion about risk is justified. Why are the S2 proponents reluctant to accept what the S1 proponents take as compelling data?

Comparative analyses with GM (single) are used in S1 papers in order to evaluate the overall stability of GM stack, including (i) interactions among transgenic proteins, (ii) interactions among transgenic and domestic proteins, and (iii) genetic/phenotypic stability. S2 proponents argue that targeted comparative analyses are an inappropriate methodology for each of these evaluations.

Concerning (i) *interactions among transgenic proteins*, Mesnage et al. (2013) argue that different combinations of transgenic proteins provoke unpredictable synergistic toxicity which will not be detected by standard comparative analyses. They suggest that a sensitive model of human cells for the systematic evaluation of every combination to which human and animals are actually exposed is a more appropriate method.

Concerning (ii) *interactions among transgenic and domestic proteins*, Agapito-Tenfen et al. (2014) argue that testing parameters separately through targeted comparative analyses is inappropriate. Londo et al. (2011), further argue that standard comparative analyses are insufficient because the constitutional, simultaneous expression of two transgenic proteins could be a high energetic cost for a GM stack. According to Londo et al., this energetic cost can change the entire metabolic set-up of the plant, which need broader analyses to be detected.

Concerning (iii) genetic/phenotypic stability, Agapito-Tenfen et al. (2014) argue that simultaneous wide-range comparisons of a large number of parameters expressed by GM stacks in comparison with single parental plants is more appropriate. They argue that such a broad, untargeted comparison enables detection of changes *prior* to any evaluation of their relevance for risk analysis is useful for detecting any issues that are novel to GM stacks. As a substantiation of this point, Ali et al. (2014) refer to recent non-targeted proteomic analyses of a variety of stacked GM maize and its GM (single) parental plants. These analyses show differences in both domestic and transgenic protein expression, differences that would go undetected in a standard analysis. Whether this influences the overall risk analyses of the GM stack remains unknown, but according to S2 proponents, the question needs to be addressed.

Following their rejection of inferences from conventional hybrids and GM (single) to GM stacks, S2 proponents thus systematically reject the appropriateness of targeted comparative analyses as a methodology for risk evaluation of GM stacks. Such methods, argue S2 authors, target exclusively risk-related issues previously known from conventional hybrids and GM (single), and are not fit to reveal any hitherto unknown GM stack-specific issues.

What we see therefore, is that although S1 and S2 proponents have the same evidence available, they make different evaluations concerning relevance and appropriateness. On the basis of these evaluations S1 and S2 proponents disagree on what constitutes a rational and science-based regulatory regime. In light of our general proposition that diverging biological background assumptions influence the S1 and S2 proponents, this is as expected. Scientific disagreement concerning regulatory regimes is therefore analyzable in terms of divergent biological background assumptions.

## Discussion

In our case study, we presented two risk analyses in which opposing conclusions are drawn from the same available evidence. We have identified two sets of implicit biological background assumptions, each correlating with a different choice of appropriate methodology and ultimately with a different evaluation of common evidence. We concluded that the disagreement is at least partially rooted at the level of biological background assumptions, and suggested that it might not be solved by mere production of more evidence. So how does this improve the debate concerning risk assessment of GM stacks?

To address this point fully, we ought to start with a general reflection about governance in case of scientific uncertainty. Following the increasing influence of different stakeholders in GM regulations, experts attitude have sometimes been that while ‘uncertainty is a ball played by stakeholders’, experts have to role of clarifying the ‘distinction between legitimate and illegitimate uncertainty. Good scientists can recognize where experiments establish certainty or leave open uncertainty’ (interview with food scientist, Levidow et al. 2007: 52). As it turns out, and as we have seen in our analysis, not all scientists agree on how to distinguish between legitimate and illegitimate uncertainty. Polarized networks of experts with divergent opinions and different interpretations of results make it possible to both contest and defend safety claims on a scientific basis.

Scientific disagreement and uncertainty have played an important role in re-setting the boundaries between science and policy (Levidow et al. 2007). For instance, disagreement on how to evaluate evidence easily leads to a faux debate over what is ‘scientifically justified’. A common approach in such debates is to accuse anyone holding an opposing view of being ‘unscientific’. Multiple examples of such accusations can be found in the scientific debate about the safety evaluation of GM plants, from ‘unscientific process’ (Fagan et al. 2015; Arjó et al. 2013) and adoption of ‘pre-conceived conclusions’ (Bøhn et al. 2012) to partial selection of evidence (Ricroch et al. 2010). Reference to the unpopularity of a position has also become commonplace as an argument for its lack of validity (Hilbeck et al. 2015; Payne 2016). Such terms do not facilitate evidence-based decision making.

Our analysis shows one possible way to frame expert’s disagreement, in the case of GM stacks. We propose that once framed, divergent evaluations of risk can contribute to the process of harmonizing global regulation of GM stacks, rather than unsettling it. In the words of Elisabeth Anderson, ‘[T]he objectivity of science demands that the background assumptions of research programs be exposed to criticism. A scientific community composed of inquirers who share the same background assumptions is unlikely to be aware of the roles these assumptions play […], and even less likely to examine these assumptions critically’ (Anderson 1995: 79).

A first crucial point is that, once the biological background assumptions are identified and exposed, it is possible to test their validity against current biological knowledge. In the basic sciences, this process of explicating hidden ontological commitments and discarding the most obsolete in relation to current knowledge has been important for paradigm shifts (Galilei 1632; Einstein 1905; Balkwill and Mantovani 2001).

A second point is that, if empirical evidence do not help in justifying one biological background assumption over another, other rational arguments can be used. For instance, by considering the implications of the choice for the final outcome of the risk evaluation and ultimately for society (see ‘inductive risk’: Douglas 2000, and this manuscript: 1.2).

Changes in GM risk regulation are mingled with value judgements as well as tensions among stakeholders. Thus constituting a very complex picture. For instance, the concept of ‘substantial equivalence’ between GM (single) and unmodified comparators has been criticized, contested, and changed in the course of the last three decades (Levidow et al. 2007). In the ‘80s and early ‘90s, the principle of ‘substantial equivalence’ was used as the scientific justification for reducing regulation of GM plants, once their chemical and nutritional composition were shown to be similar to the unmodified counterpart (OECD 1993; WHO/WTO 1991; European Commission (EC) 1997). Later on, however, in correlation with social and political pressures from NGOs and consumer associations, experts expressed split opinions about the validity of the principle. The use of mere targeted chemical composition as a criterion to establish similarity between GM plants and their conventional counterparts was famously criticized as ‘unscientific’ in authoritative scientific journals (Millstone et al. 1999). The firing of controversies resulted into a transatlantic split in GM food regulation, with USA favoring the ‘substantial equivalence’ approach and Europe requiring a more comprehensive safety protocol before licensing GM plants for market introduction (Levidow et al. 2007). Global regulation of GM plants was eventually re-harmonized by acknowledging that the original definition of ‘substantial equivalence’ was scientifically unsatisfactory, and changing it into ‘the comparative approach’ of risk assessment, which includes immune-toxicological tests and analysis of the insert’s stability over generations (Codex Alimentarius Commission (CAC) 2003; European Food and Safety Agency (EFSA) 2004).

We consider the discourse on ‘substantial equivalence’ between single GM and conventional plants as exemplary of how ‘scientific soundness’ is a variable concept, and how extra-evidential components (both socio-political value judgments and biological background assumptions) are significant for determining it. Our take is that, while scientific debates are important in the dialogue on risk regulation, they need to primarily create, rather than obscure, transparency. The first step toward transparency is to become aware of biological background assumptions that are built into technical concepts, and to define how they relate to the scientific state of the art. Had this been done in concomitance with the initial proposal of ‘substantial equivalence’ - based regulation in the early ‘90s, stakeholders might have been better informed, and the final harmonization reached earlier.

To our knowledge, this is the first analysis of biological background assumptions in the discussion on GM stacks regulation, and GM plants in general. We suggest that it can be the initial step for a more self-aware scientific debate. A scientific debate more adequate to inform governance.

We suggest that our approach to the discussion about GM stacks could be a general framework for instances where risk evaluation is underdetermined by evidence. In sum, this consists of (a) acknowledging that biological background assumptions are part of risk assessment, even if rarely explicated; (b) identifying which biological background assumptions are at play and how; (c) finding empirical and/or argumentative reasons (for instance through ‘inductive risk’) to adopt one set of assumptions rather than another.

## Abbreviations

**GM (single)**: parental single genetically modified plant
**GM stack**: stacked genetically modified plant

## Footnotes

1 Note that GM Stacks can be obtained also by multiple transgenic modification. These are not object of this paper, since there is no disagreement that such types of GM Stacks are GM plants to all effects.

2 With our terminology, we indicate different relative positions in an argumentative spectrum, rather than extreme positions. For instance, “process equivalence” does not imply a supposition of total equivalence, rather a supposition of significantly more similarity of biological processes compared to “process variability”.

3 In the following, “phenotype” refers to compositional, nutritional, agronomic traits and level of transgene expression.

4 Note that by “process” here, Steiner et al. are referring to the methodological process, which is identical in conventional breeding and GM stacking.

5 See for instance Einstein’s treatment of kinematics (Einstein 1905) and Galilei’s treatment of the tower argument (Galilei 1632). In biology, see the causal interpretation of correlation data between inflammation and cancer (Balkvill and Mantovani. 2001).

## References

[CR1] Agapito-Tenfen SZ, Vilperte V, Benevenuto RF, Rover CM, Traavik TI, Nodari RO (2014). Effect of stacking insecticidal cry and herbicide tolerance Epsps transgenes on transgenic maize proteome. BMC Plant Biol.

[CR2] Ali S, Ben E, Madi ZE, Hochegger R, Quist D, Prewein B, Haslberger AG, Brandes C (2014). Mutation scanning in a single and a stacked genetically modified (GM) event by real-time PCR and high resolution melting (HRM) analysis. Int J Mol Sci.

[CR3] Althaus CE (2005). A disciplinary perspective on the epistemological status of risk. Risk Anal.

[CR4] Anderson E (1995). Feminist epistemology: An interpretation and a defense. Hypatia.

[CR5] Arjó G, Portero M, Piñol C, Viñas J, Matias-Guiu X, Capell T, Bartholomaeus A, Parrott W, Christou P (2013). Plurality of opinion, scientific discourse and pseudoscience: An in depth analysis of the Seralini et al. study claiming that roundup™ ready corn or the herbicide roundup™ cause cancer in rats. Transgenic Res.

[CR6] Balkwill F, Mantovani A (2001). Inflammation and cancer: Back to Virchow?. Lancet.

[CR7] Bøhn, Thomas, Raul Primicerio, and Terje Traavik. 2012. The German ban on GM maize MON810: Scientifically justified or unjustified? Environmental sciences Europe 24 (1). Environmental sciences Europe: 1. doi:10.1186/219047152422.

[CR8] Carver R, Waldahl R, Breivik J (2008). Frame that gene. A tool for Analysing and classifying the communication of genetics to the public. EMBO Rep.

[CR9] Codex Alimentarius Commission (CAC). 2003. Joint WHO/WHO Food Standards Programme, Codex Committee on General Principles, Appendix V, Working Principles for Risk Analysis. Rome, 30 June to 7 July. Available at www.WHO.org/input/download/report/47/Al0312ae.pdf.

[CR10] Douglas H (2000). Inductive risk and values in science. Philos Sci.

[CR11] Einstein A (1905). On the electrodynamics of moving bodies (Zur Elektrodynamik Bewegter Körper). Ann Phys.

[CR12] Einstein, A.1936. Physics and reality. In: Ideas and opinions, ed. Albert Einstein, 290–312. New York: Crown Publishers Inc.

[CR13] Ellis G, Silk J (2014). Scientific method: Defend the integrity of physics. Nature.

[CR14] European Commission Directorate for Health and Consumers. 2010. Evaluation of the EU legislative framework in the field of GM food and feed. https://ec.europa.eu/food/sites/food/files/…/gmo_repstud_2010_report_eval-gm.pdf. Accessed 03 July 2017.

[CR15] European Commission (EC). 1997. Regulation 258/97/EC of 27 January 1997 Concerning Novel Foods and Novel Food Ingredients. Official Journal of the European Communities L43 (14 February): 1–6.

[CR16] European Food and Safety Agency (EFSA). 2004. Guidance document for the risk assessment of genetically modified plants and derived food and feed by the Scientific Panel on Genetically Modified Organisms (GMO). Available at http://www.efsa.europa.eu/en/efsajournal/pub/99.

[CR17] European Food and Safety Agency (EFSA). 2007. Guidance document for the risk assessment of genetically modified plants containing stacked transformation events by the scientific panel on genetically modified organisms (GMO). EFSA J 5: 1–5 http://onlinelibrary.wiley.com/doi/10.2903/j.efsa.2007.512/abstract. Accessed 13 May 2016.

[CR18] Fagan, John, Terje Traavik, and Thomas Bøhn. 2015. The Seralini affair: Degeneration of science to re-science? Environmental sciences Europe 27 (1). Springer Berlin Heidelberg: 19. doi:10.1186/s12302-015-0049-2.

[CR19] FAO/WHO. 1991. WHO expert co/WHO expert consultation, strategies for assessing the safety of foods produced by biotechnology. Rome: FAO.

[CR20] Funke, Todd, Huijong Han, Martha L Healy-Fried, Markus Fischer, and Ernst Schönbrunn. 2006. Molecular basis for the herbicide resistance of roundup ready crops. Proc Natl Acad Sci U S A 103 (35): 13010–13015. doi:10.1073/pnas.0603638103. 10.1073/pnas.0603638103 PMC1559744 16916934

[CR21] Galilei, Galileo. 1615. Considerations on the Copernican opinion. In the Galileo affair: A documentary history, ed. Maurice Andrea Finocchiaro, 70–86. University of California Press, 1989.

[CR22] Galilei, G. 1632. Dialogue concerning the two chief world systems. Ed. Drake (trans) & Gould. New York: Modern Library.

[CR23] Hartley S, Gillund F, van Hove L, Wickson F (2016). Essential features of responsible governance of agricultural biotechnology. PLoS Biol.

[CR24] Hermansson, Helene. 2012. Defending the Conception of ‘Objective Risk.’ Risk Anal 32 (1): 16–24. doi:10.1111/j.1539-6924.2011.01682.x. 10.1111/j.1539-6924.2011.01682.x 21919929

[CR25] Hilbeck A, Binimelis R, Defarge N, Steinbrecher R, Székács A, Wickson F, Antoniou M (2015). No scientific consensus on GMO safety. Environ Sci Eur.

[CR26] Jasanoff S (2005). Designs on nature: Science and democracy in Europe and the United States.

[CR27] Kok, Esther J., Jan Pedersen, Roberta Onori, Slawomir Sowa, Marianna Schauzu, Adinda De Schrijver, and Teemu H. Teeri. 2014. Plants with Stacked Genetically Modified Events: To Assess or Not to Assess? Trends in Biotechnology 32 (2). Elsevier Ltd: 70–73. doi:10.1016/j.tibtech.2013.12.001. 10.1016/j.tibtech.2013.12.001 24418332

[CR28] Kramer C, Brune P, McDonald J, Nesbitt M, Sauve A, Storck-Weyhermueller S (2016). Evolution of risk assessment strategies for food and feed uses of stacked GM events. Plant Biotechnol J.

[CR29] Kuhn, Thomas. 1970. The structure of scientific revolutions. 2nd edn.: University of Chicago Press, Chicago

[CR30] Kuhn, Thomas. 1973. Objectivity, value judgement, and theory choice. In the essential tension, 320-39. University of Chicago Press, Chicago, 1977.

[CR31] Levidow L, Murphy J, Carr S (2007). Recasting ‘substantial equivalence’: Transatlantic governance of GM food. Sci Technol Hum Values.

[CR32] Londo, J P, M a Bollman, C L Sagers, E H Lee, and L S Watrud. 2011. Changes in Fitness-Associated Traits due to the Stacking of Transgenic Glyphosate Resistance and Insect Resistance in *Brassica napus* L. Heredity 107 (4). Nature Publishing Group: 328–37. doi:10.1038/hdy.2011.19. 10.1038/hdy.2011.19 PMC3182500 21427753

[CR33] Longino HE (1990). Science as social knowledge:Values and objectivity in scientific tradition and change.

[CR34] Mesnage R, Clair E, Gress S, Then C, Székács A, Séralini GE (2013). Cytotoxicity on human cells of Cry1Ab and Cry1Ac Bt insecticidal toxins alone or with a glyphosate-based herbicide. J Appl Toxicol.

[CR35] Miller G, Wickson F (2015). Risk analysis of nanomaterials: Exposing Nanotechnology's naked emperor. Review of Policy Research.

[CR36] Millstone E, Brunner E, Mayer S (1999). Beyond ‘Substantial Equivalence’. Nature.

[CR37] OECD. 1993. Safety evaluation of foods derived by modern biotechnology: Concepts and principles. Paris: Organisation for Economic Cooperation and Development.

[CR38] Payne, Jack M. 2016. Standing up for Scientific Consensus. Nature Biotechnology 34 (1). Nature Publishing Group: 23–24. doi:10.1038/nbt.3454. 10.1038/nbt.3454 26744971

[CR39] Pilacinski, W., A. Crawford, R. Downey, B. Harvey, S. Huber, P. Hunst, L. K. Lahman, et al. 2011. Plants with genetically modified events combined by conventional breeding: An assessment of the need for additional regulatory data. Food and chemical toxicology 49 (1). Elsevier ltd: 1–7. doi:10.1016/j.fct.2010.11.004. 10.1016/j.fct.2010.11.004 21074592

[CR40] Portier, Christopher J, Bruce K Armstrong, Bruce C Baguley, Xaver Baur, Igor Belyaev, Robert Bellé, Fiorella Belpoggi, et al. 2016. Differences in the carcinogenic evaluation of glyphosate between the International Agency for Research on Cancer ( IARC ) and the European food safety authority ( EFSA ). J Epidemiol Community Health 0 (0): 1–5. doi:10.1136/jech-2015-207005. 10.1136/jech-2015-207005 PMC4975799 26941213

[CR41] Que Q, Chilton M-DM, de Fontes CM, He C, Nuccio M, Zhu T, Wu Y, Chen JS, Shi L (2010). Trait stacking in transgenic crops: Challenges and opportunities. GM Crops.

[CR42] Reichenbach, H. 1927. “Philosophie Der Raum-Zeit-Lehre.” In The Philosophy of Space and Time, ed. Hans Reichenbach and Jung Freund. New York: Dover Publication, Inc.

[CR43] Ricroch A, Bergé JB, Kuntz M (2010). Is the German Ggsuspension of MON810 maize cultivation scientifically justified?. Transgenic Res.

[CR44] Sawyer, Suzanna. 2015. Crude contamination: Law, science, and indeterminacy in Ecuador and beyond. In: Subterannean Estates: Life worlds of oil and gas. Eds Hannah Appel, Arthur Mason, Michael watts, 126-146. New York: Cornell University press.

[CR45] Steiner H-Y, Halpin C, Jez JM, Kough J, Parrott W, Underhill L, Weber N, Curtis Hannah L (2013). Evaluating the potential for adverse interactions within genetically engineered breeding stacks. Plant Physiol.

[CR46] Stotz K, Griffiths PE, Knight R (2004). How biologists conceptualize genes: An empirical study. Studies in History and Philosophy of Science Part C :Studies in History and Philosophy of Biological and Biomedical Sciences.

[CR47] Stump D (2015). Conceptual change and the philosophy of science alternative interpretations of the a priori.

[CR48] VKM. 2016. Comments from The Norwegian Scientific Committee for Food Safety ( VKM ) GMO Panel on the application for maize MON 87427 x MON 89034 x MIR162 x NK603 www.vkm.no/dav/3d8bd3cadf.pdf. Accessed 13 May 2016.

[CR49] Weber N, Halpin C, Hannah LC, Jez JM, Kough J, Parrott W (2012). Crop genome plasticity and its relevance to food and feed safety of genetically engineered breeding stacks. Plant Physiol.

[CR50] Wickson F, Wynne B (2012). Ethics of science for policy in the environmental governance of biotechnology: MON810 maize in Europe. Ethics, Policy & Environment.

